# Relevance relations for the concept of reproducibility

**DOI:** 10.1098/rsif.2013.1030

**Published:** 2014-05-06

**Authors:** H. Atmanspacher, L. Bezzola Lambert, G. Folkers, P. A. Schubiger

**Affiliations:** 1 Collegium Helveticum, Zurich, Switzerland; 2 Institute for Frontier Areas of Psychology, Freiburg, Germany; 3 Department of English, University of Basel, Switzerland

**Keywords:** reproducibility, relevance, complexity

## Abstract

The concept of reproducibility is widely considered a cornerstone of scientific methodology. However, recent problems with the reproducibility of empirical results in large-scale systems and in biomedical research have cast doubts on its universal and rigid applicability beyond the so-called basic sciences. Reproducibility is a particularly difficult issue in interdisciplinary work where the results to be reproduced typically refer to different levels of description of the system considered. In such cases, it is mandatory to distinguish between more and less relevant features, attributes or observables of the system, depending on the level at which they are described. For this reason, we propose a scheme for a general ‘relation of relevance’ between the level of complexity at which a system is considered and the granularity of its description. This relation implies relevance criteria for particular selected aspects of a system and its description, which can be operationally implemented by an interlevel relation called ‘contextual emergence’. It yields a formally sound and empirically applicable procedure to translate between descriptive levels and thus construct level-specific criteria for reproducibility in an overall consistent fashion. Relevance relations merged with contextual emergence challenge the old idea of one fundamental ontology from which everything else derives. At the same time, our proposal is specific enough to resist the backlash into a relativist patchwork of unconnected model fragments.

## 1. Introduction

The reproducibility of research results is one of the key cornerstones of scientific methodology—a gold standard for science as it were. According to Tetens [1, p. 593],

reproducibility means that the process of establishing a fact, or the conditions under which the same fact can be observed, is repeatable. … The requirement of reproducibility is one of the basic methodological standards for all sciences claiming lawlike knowledge about their domain of reference. In particular, reproducibility is an inevitable requirement for experiments in the natural sciences: each experiment must be repeatable at any time and at any place by any informed experimentalist in such a way that the experiment takes the same course under the same initial and boundary conditions. The reproducibility of an experiment in the natural sciences includes the reproducibility of experimental setups and measuring instruments.

Ideally, research results are only worthy of attention, publication and citation if independent researchers can reproduce them. But for much of the current scientific literature reproducibility has become a serious problem. In areas as diverse as social psychology [2], biomedical sciences [3], computational sciences [4] or environmental studies [5]^1^ researchers not only find serious flaws in reproducing published results but also launch initiatives to counter what they regard as dramatically undermining scientific credibility. Whether owing to simple error, misrepresented data or sheer fraud irreproducibility corrupts both intra-academic interactions based on truthfulness as well as the science–society link based on the trustworthiness of scientific evidence.

The present essay addresses one specific question concerning the problem of reproducibility—the question of which properties of a system in a given context are actually relevant for its description and should thus be taken as the target to be reproduced. After some more general remarks in §2, we first document problems with reproducibility in basically three areas (§3): inanimate matter (physics, chemistry), living organisms (biomedical sciences) and mental processes (psychology, cognitive science, consciousness studies). It is evident that, according to the authors' professional profiles, many areas of human knowledge, such as economics, law, political science, history and others, had to be left out of consideration in this article.

In §4, it is shown how appropriate relevance criteria relative to particular situations and their contexts can be identified. Based on previous work in the philosophy of science and in communication theory, we propose the concept of a *relevance relation* as an effective tool to distinguish relevant from irrelevant features of a system at a given level of description. A relevance relation is a relation between the level of complexity at which a system is considered and the granularity of its description. For the behaviour of highly complex mental systems, a description in terms of their elementary physical constituents is arguably beside the point, while for simple systems of classical mechanics a discussion of semantic or pragmatic aspects seems absurd. For each level of complexity, relevance relations distinguish particular system properties within a suitably coarse-grained description. This idea finds most powerful applications in interdisciplinary research, where different levels of description must typically be considered together.

The essay concludes with some philosophical perspectives proposing a somewhat unorthodox move in the long-standing realism debate. Beyond the concept of reproducibility, relevance relations are also important for proper explanations of observed phenomena. This entails an ‘explanatory relativity’, which expresses that explanations are generally relative to the level of complexity at which a system is considered in a particular context. Pushing this relativity even further, we may speculate about an ‘ontological relativity’, as introduced by Quine. It departs from the centuries-old conviction of one fundamental ontology to which everything can be ultimately reduced. At the same time, well-defined relevance relations allow us to resist an unsatisfactory relativism of arbitrarily connected (or unconnected) beliefs and opinions.

## 2. Why reproducibility, and how?

From an ontological point of view, the idea of reproducibility derives from the presumption of *ontically given*, *invariant*, *stable structures of nature* giving rise to lawful behaviour. In contrast to sense or introspective data, the ontic structures are assumed to be universal rather than particular. Insofar as empirical data derive from their ontic, invariant origin, any proper empirical knowledge (perception, observation or measurement) based on those data should reveal their underlying structure. As a consequence, it should be possible to reproduce empirical data indicative of the same invariant structure independent of where, when or by whom the perception, observation or measurement is conducted.

Reproducibility is regarded as a central methodological criterion of the sciences.^2^ If an empirical observation cannot be reproduced, it will in general be ignored, disregarded or even declared fraudulent—irreproducible results do not belong to the established body of scientific knowledge. Nevertheless, the reproducibility of an empirical result is only necessary, not sufficient for its acceptance in the sciences. An essential additional condition is the consistent incorporation and interpretation of reproduced results in a theoretical framework.

To reproduce an empirical result means to observe it under circumstances identical (as identical as possible, that is) with those which led to its preceding observations. This presupposes that the relevant circumstances must be known and controlled to such an extent that they can be re-established in future attempts to reproduce an observation. If the circumstances are known well enough, the aspect of control is typically guaranteed by suitable laboratory designs. A proper experimental set-up enables a precise and reproducible observation of a selected feature of a system.

Today it is a truism that experiments do not only ‘reveal’ features of nature but also play a ‘constructive’ role—most prominently, experimental set-ups in quantum physics decide whether a system appears with wave-like or particle-like features.^3^ However, this does not mean that arbitrarily chosen features can be created irrespective of the system considered. In this sense, the invariant stable structures mentioned above are an essential part of the image of nature that science presents. They are indispensable for the rigorous mathematical formalizations developed by the sciences and they limit the constructive potential of empirical arrangements.

Experiments provide answers only to questions for which they are properly designed. This is sometimes expressed by the notion of a ‘Procrustes strategy’ governing laboratory-based science.^4^ More generally, and most obviously outside the laboratory, there are always contexts that cannot be controlled but that can be disregarded if they are irrelevant for the question to be answered. It is part of the art of empirical science to design experiments in such a way that the context of a particular question is mapped into relevant experimental conditions as precisely as possible. For the reproducibility of a result, it does not matter if irrelevant conditions vary: only the relevant conditions must be kept fixed. This raises the question of how the relevance of a condition can be determined in the first place, and we argue below (§4) that it can only be determined for a concrete situation and its contexts.

Many large-scale systems (e.g. geophysics or astrophysics) do not permit any active control of empirical observations. Such situations are examples of complex physical systems without well-controlled boundary conditions, entailing the loss of precise control. In subtler ways, this may also be the case under laboratory conditions, for instance, in intrinsically unstable dynamical systems (‘chaos’) or structural instabilities of materials, fluids, plasmas, etc. It becomes an even more challenging problem for living organisms, distinguished by intrinsic behavioural autonomy, and in those areas of the life (or biomedical) sciences where rigorously fixed laboratory conditions are often unrealistic or even nonsensical. Nevertheless, a huge body of important and sophisticated knowledge has been collected for such systems.

In addition, the rapidly increasing power of computational devices today allows us to study the behaviour of complex systems by numerical simulation (rather than empirically). This applies to areas of science such as computational mathematics, climate simulations, ‘omics’-research, neuroscience and other areas of big-data science (e.g. [4,15,16]). Apart from the problems of reproducibility as such, critical voices have remarked that large-scale simulations, even if they yield reliable numerical results, will not automatically lead to improved understanding and insight.

## 3. Examples

### 3.1. Inanimate matter

A special form of reproducibility in cases where observables can be measured quantitatively posits that the measured value of an observable must be consistent with the values obtained by previous measurements. Precise identity is not required—it is well known that experimental results always have (epistemic) measurement errors. For this reason, a limit for an admissible scatter of individual results is usually set (by convention) beyond which results are not considered as successfully reproduced.

A pertinent example is the motion of a rigid body, for instance its free fall in a gravitational field. Typical relevant observables in this case are its position and momentum. These and other observables of mechanics are called ‘canonical’—they satisfy the criteria of a so-called ‘symplectic structure’. If there were no measurement errors, the time that it takes for a rigid body to fall a certain distance in a particular gravitational field would be precisely identical in each repetition of the experiment. The reason is that the laws of gravity governing the motion of the body are *deterministic*.

In the realistic case of measurement errors, it is necessary to apply statistics; in many simple situations, the standard deviation of a distribution of measured values around a mean serves to distinguish results consistent with the expected error distribution from those which are inconsistent (so-called ‘outliers’). A result is considered reproduced if it is consistent with the expected distribution. This presupposes that the error distribution of measured results is known, e.g. as a normal (Gaussian) distribution. Moreover, it must be ascertained that the measured results are not obscured by systematic errors or must be corrected for such errors if known.

There is a great deal of properties of falling bodies that are plainly irrelevant for the impact of gravity, for instance, their colour, shape, texture, etc. Numerous facts do not contradict predictions based on the laws of gravity but are not entailed by them. For instance, the laws of gravity predict the point where the body will fall, yet they leave open whether it will rebound, remain motionless or break into pieces. It is also irrelevant whether a text message is written on the body's surface or hidden in its interior, which might be highly informative for an ‘initiated’ observer.

There are cases in which a system consists of many individual bodies so that it becomes extremely tedious, or impossible, to follow all their trajectories individually. Such many-body (or many-particle) systems have been studied in statistical physics for a long time, and it has turned out that the canonical observables of each single particle are, in a certain sense, irrelevant for the behaviour of the system as a whole. Thermal systems are examples of this situation and a whole new set of observables has been defined for them in thermodynamics: temperature, pressure, entropy and so on.

These thermodynamic observables can be related to the moments of distributions over the canonical observables of mechanics and reflect the relevant properties for ensembles of particles. However, they are not distributions of errors but of actual values. From the distribution of the momenta of all individual particles, one can calculate the mean kinetic energy, which is proportional to the temperature of the gas. Although they can be based on dispersive statistical distributions of mechanical observables, thermodynamic observables themselves are dispersion-free. Positions and momenta of particular gas molecules are irrelevant for the temperature of the gas, as long as the momenta for the full ensemble yield a (Maxwell) distribution of the proper form.

Hydrodynamics, solid-state physics, chemical kinetics and other areas of science can be described at this level of sophistication. As for the falling body, higher level contingent contexts and conditions often are irrelevant for a proper description. For instance, whether a particular gas has the capacity to kill oxygen-breathing organisms is irrelevant for its temperature, whether crystals are optically active is irrelevant for their weight or whether liquids are drugs or perfumes is irrelevant for their viscosity.

### 3.2. Living organisms

The two basic kinds of reproducibility for results in inanimate systems are: (i) sharp, dispersion-free (ontic) values which could be repeated identically if there were no measurement errors and (ii) distributions of some canonical form (e.g. Gauss, Maxwell, Boltzmann) which are to be reproduced whenever deterministic laws for individual systems are unknown or intractable and statistical laws need to be considered.^5^ (These preconditions may lose their rigour or may even become meaningless in complex systems far from thermal equilibrium, even though those systems are still inanimate.)

Living organisms operate far from equilibrium and exhibit autonomous reactions to environmental stimuli, which are not governed by universal (deterministic or statistical) laws as in physics. Even if the relevant observables are individually measurable, they typically give rise to distributions without any theoretically known (canonical) form. Their variability does not refer to measurement errors alone but also comprises a natural variability of ontic values. The overall distribution of measurement results is then a convolution of the natural distribution with the error distribution. It is obvious that this can entail all sorts of complications for statistical analyses, the choice of a proper statistical model and the ‘substantive context’ [17] of a study in general. In the following, we will point to some selected problem areas which have been found particularly challenging.

One significant area in this respect is the reproducibility of results from animal studies in preclinical research. Apart from the fact that the standards for reports of such research are surprisingly often ignored [18–20], even well-documented studies show a severe lack of reproducibility [21]. In typical attempts to standardize the animal species for the experiments, homogeneous inbred strains, minimalistic cage environments (husbandry) or narrow group selection (e.g. male, same age) are used, assumed to be the ideal guarantee for reproducible results. However, this procedure weakens the natural distribution of the species by replacing it with an artificial substitute, which arguably destabilizes their behaviour, including reactions to drugs. Würbel [22] has coined the notion of a ‘standardization fallacy’ for such situations (see also [23])—actually a better term would be ‘homogenization fallacy’: a proper standardization for experiments with living organisms should not overcontrol the sample but reflect its natural distribution.

System features with ontic variability, typical for living organisms, give rise to a natural distribution with non-vanishing dispersion. In this case, it is crucial to use this distribution for controlled experiments rather than overly homogenized substitute distributions with spuriously minimized dispersion, which may be irrelevant for the actual behaviour of the system. It is tempting to speculate that the limited reproducibility of results under extremely homogeneous conditions is not only due to the fact that these conditions simply cannot be precisely reproduced, but that the artificially small variability itself has a destabilizing effect. Similar situations are known in ecosystems: reducing the biodiversity of such systems can increase their vulnerability against small perturbations dramatically [24,25].

Another striking kind of variability, which instigated doubts about reproducible results, has been unveiled by Anderson [26]. In contrast to the long unquestioned assumption that mental states have stable neural correlates, his meta-analysis of 1469 functional magnetic resonance imaging studies in 11 domains of cognitive tasks revealed that various brain regions are typically involved in tasks across task domains. Although the author has much more to say about this observation, one conclusion is that the picture of a simple one-to-one or even many-to-one correspondence between neural states and mental states is fatuous—one has to expect many-to-many correlations, and thus a much more complex dependence of neural correlates on cognitive tasks or vice versa.

As a consequence of these and other recent insights, reproducibility has become a controversial issue in biomedical and life sciences. A report in the *New Yorker* by Lehrer [27] prompted intense discussion, e.g. in *Nature* [28] or in the prestigious *Journal of Statistical Physics* [29]. The journal *Science* devoted a special issue to the topic in December 2011. The journal *PLoS ONE* launched a ‘reproducibility initiative’ in 2012,^6^ and three prominent psychology journals jointly established a ‘reproducibility project’ recently.^7^

The *Journal of Negative Results in Biomedicine*, with its editorial office at Harvard Medical School, was launched in 2002 to overcome a well-known deficit in publication policy. They encourage the submission of experimental and clinical research falling short of demonstrating improvements over current state-of-the-art or failures to reproduce previous results. Better knowledge about such (and other) negative results is also important for sound meta-analyses of grouped studies, which often suffer from a publication bias towards positive results. An extensive report on reproducible research as an ethical issue is owing to Thompson & Burnett [30].

A particularly interesting development is the emergence of the field of ‘forensic bioinformatics’. The pioneers of this field, Baggerly and Coombes (M.D. Anderson Cancer Center, Houston) describe their approach as the attempt to reconstruct what might have gone wrong in particular experiments and their data analysis if the reported results are inconsistent with what their authors claimed to have done. In a spectacular case (the so-called ‘Potti case’), more than 1500 hours of tedious work [31] proved that a series of trivial errors and mistakes led to serious ramifications, including clinical studies under false preconditions and the termination of their author's employment.

Increasingly sophisticated technologies in biomedical sciences and in computational data analysis seduce scientists to produce enormous amounts of data (‘big data’) in so-called high-throughput experiments. These avalanches of data are often not collected to test hypotheses or, ultimately, provide evidence for certain models or theories—they are collected because it is technically possible to collect them. The hope is that tricky ways of ‘data mining’ will unveil trends, patterns and correlations generating insight. However, it is far from evident whether this hope is justified. Modifying a famous dictum by Wittgenstein,^8^ it may be more realistic to conceive of science as ‘the battle against the bewitchment of our intelligence by data’.

### 3.3. Mental processes

The notion of mental processes (such as emotion, cognition or decision-making) refers to systems that exceed merely physical, chemical or biological behaviour. For instance, they may produce, interpret or understand meaning, and they may intend and perform actions. It is often difficult (and controversially discussed) to delineate mental processes conclusively from their material (e.g. neural) correlates. However, it is fairly uncontroversial that semantics is no explicit topic of investigation in the traditional natural sciences. By contrast, psychology, cognitive science, philosophy of mind, linguistics, literary studies and other areas have developed a variety of approaches to address the concept of meaning.

Psychology is one main discipline in which mental processes of human subjects are studied. Although some areas of psychology try to find ways to analyse mental processes in terms of variables that can be numerically evaluated (quantified), much of psychology relies on the detection of patterns rather than numbers. Generally speaking, patterns are objects of classifications, which, for instance, are often used for the analysis of surveys and questionnaires. They derive from methods of multivariate statistics, most commonly cluster analyses, factor analyses or principal component analyses.^9^

If such patterns are identified, *they* are the targets of reproducibility, not particular responses to particular questions or numerical valuations of variables. For instance, one can look at distributions of patterns in different cultural contexts and check whether they are reproducible across cultures. Or one can study psychiatric symptoms and check whether they are restricted to diagnosed patients or occur more broadly in the general population as well. The latter case has received increasing attention as the so-called ‘continuum hypothesis’ [34]. Its confirmation requires reproducible patterns across different subsamples of subjects (for a recent study see [35]).

A recently much disputed area exhibiting problems with reproducibility is social priming, the impact of unconscious cues on behaviour [36]. Stimulated by an open e-mail in which Nobel prize winner Daniel Kahneman recently urged researchers to restore the shaken credibility of their field,^10^ a large-scale study of direct replications concerning 13 different kinds of social priming were conducted in 36 independent samples and settings. This heroic effort led to a 51-author paper [37], which reports that 10 out of 13 kinds of social priming have been reproduced consistently, yet with considerable variations in effect size, across laboratories.

Concerning *agency*, one outstanding example for fallacious arguments based on irrelevant features is the free-will discussion in and after the 1990s, the ‘decade of the brain’.^11^ A remarkable number of neuroscientists and neurophilosophers declared the freedom of will and agency as illusions, basically for the grossly misleading reason [38] that brains are deterministic machines, no matter whether their operations are consciously experienced or remain unconscious. Bennett & Hacker [39] emphasized one major flaw in such arguments as the ‘mereological fallacy’, confusing the behaviour of wholes (experiencing subjects) with the behaviour of their parts (organs, neurons, genes). Neurons and synapses tell us as much about the freedom of will and agency that persons may or may not execute as blood pressure or heart rate tell us. Meanwhile, prominent ringleaders of the no-free-will campaign backpedalled or fell into silence.

In the wide field of disciplines concerned with language and communication, such as linguistics (particularly semantics and pragmatics), hermeneutics, literature and literary studies, a key issue for reproducibility is the *meaning* of a text. Its production or reproduction depends on historical, cultural, or more broadly situational contexts. As the conventionalized medium of language is used by individual subjects, complete reproducibility is clearly impossible. Just as thoughts can never be precisely or exhaustively conveyed through words, the intended meaning of an utterance or text is never congruent with the meanings construed by its recipients; nor is it comprehensively recoverable in pre-linguistic form by the speakers themselves. Rather than just reproduced, meaning is actively produced and its production underlies translation and adaption on multiple levels. This applies for oral communication, but especially for textually recorded speech.

In the production of literature and other art forms, the notion of imitation plays a central role. According to the classical Aristotelian definition, art imitates nature and reality (or human affairs) either in the form of a direct imitation (*mimesis*^12^) or of a mediated or narrated imitation (*diegesis*). Yet even in its most imitative/reproductive form (realism), art aims to present a condensed and structured imitation of reality (in Aristotelian terms, it offers a complete or unified action) that conveys the human condition in a meaningful way.

Besides the more broadly imitative character of art in relation to reality, in the world of letters the concept of reproducibility manifests itself most poignantly in the practice of literary imitation, a text that uses an extant well-known literary work as a model for imitation. It may do so either with an attitude of nostalgic reverence toward an irrecoverable past (a literary practice called *imitatio*) or in a more playful, irreverent or critical fashion (parody). Despite the basic imitative character of these practices, both variants essentially depend on the incompatibility of imitation and model that may be captured by the phrase *repetition with a difference* [42].

The same principle that governs tangential issues of reproducibility in literary production also applies in the field of hermeneutics and historicist approaches to texts. The ‘difference’ in this case concerns the problem that the cultural context from which the critical reader approaches the text in question does not coincide with the situational context in which the text was written. The difficulty then lies in doing justice to the cultural conditioning of a given work of art and its participation in a contingent set of historical relations that to a large extent remains irrecoverable, rendering accurate reproduction impossible. Addressing this problem requires, first of all, recognizing it by acknowledging the cultural determinedness of any particular viewpoint and the limits of communication this engenders; secondly, it involves the active attempt to gain more fine-grained insights into a particular cultural field by reintegrating the literary text into a wider web of contemporary discourses so as to recharge the text with its original cultural energy [43].

It is largely irrelevant for the issue of meaning how printer colours are chemically composed, or which statistics governs the distribution of letters in the text.^13^ Yet, depending on the specific kind of analysis conducted, questions such as whether a text was originally circulated in print or handwritten, or what spelling conventions were used, may gain relevance in attempts to reproduce historically determined meaning. Accordingly, such issues have been given increasing weight in literary studies or editorial practice, together with consideration of hegemonic discourse, fashions, literary genres, the politics of the publishing system and other factors in the genesis of a text.

## 4. Relevance relations

### 4.1. Relevant explanations and explanatory relativity

When Willie Sutton was in prison, a priest who was trying to reform him asked him why he robbed banks. ‘Well’, Sutton replied, ‘that's where the money is’. Garfinkel [44, p. 21], who introduces his chapter on ‘explanatory relativity’ with this example, argues that the palpable misfit of question and answer arises because Sutton and the priest have different sets of alternatives in mind, different ‘contrasts’ as it were. The priest wants to know why Sutton goes robbing rather than leading an honest life, whereas Sutton explains why he robs banks rather than gas stations or grocery stores.

Another example of Garfinkel's explanatory relativity is illustrated by van Fraassen [11, p. 125], quoting Hanson: ‘Consider how the cause of death might have been set out by a physician as “multiple haemorrhage”, by the barrister as “negligence on the part of the driver”, by a carriage-builder as “a defect in the brakeblock construction”, by a civic planner as “the presence of tall shrubbery at that turning”'. All these different ways to ‘explain’ the death of one particular person in one particular incident are examples of different relevance relations, corresponding to different contrast classes.^14^

On the accounts of Garfinkel and van Fraassen (and others), explanations are not only relationships between theories and facts: they are three-place relations among theories, facts and contexts. Relevance relations as well as contrast classes are determined by contexts that have to be selected and are not themselves part of a scientific explanation. This assertion is an essential piece of van Fraassen's anti-realist stance of ‘constructive empiricism’. Science tries to explain the structure of the phenomena in the world (possibly all of them), but it does not determine which parts of that structure are salient in particular situations.

The importance of context in determining relevance is also emphasized in ‘relevance theory’, a pragmatic theory of communication formulated by Sperber & Wilson [45]. Relevance theory aims to explain how the receiver of a message infers the meaning intended by the sender based on the evidence provided in the associative *context* of the message.^15^ The same syntactic message can have very different meanings in different contexts. According to relevance theory, the receiver's proper interpretation of a message then depends on his or her ability to establish maximal relevance for the message within the specific context in which it is sent, while keeping the processing effort minimal.

Relevance theory also offers a useful characterization of rhetorical figures, such as metaphor, hyperbole (exaggeration) and irony, which have proved to be notoriously difficult to define without falling prey to simplistic reduction. Wilson & Sperber [48] describe them as parts of a spectrum of communicative modes presenting instances of relevance optimization. Instead of the classic definition of irony as an expression that means the opposite of what is said, they define irony as an act of quoting that implies a personal attitude to the quoted expression, which needs to be inferred correctly in order to optimize relevance. This illustrates the creative element involved in meaning production and is related to (if not identical with) the choice of the proper contrast class in van Fraassen's and Garfinkel's accounts.

### 4.2. Descriptive granularity versus level of complexity

Explanatory relativity (Garfinkel) expressed by relevance relations (van Fraassen) can be considered as a template for discussing the issue of reproducibility. Features that are relevant for a proper explanation of some observation should have a high potential to be also relevant if that observation is to be robustly reproduced. But which properties of systems and their descriptions may be promising candidates for the application of such relevance relations? As the first step toward more refined approaches to be developed in the future, we propose that relevance relations characterize how the *granularity* (coarseness) of a description is related to the level of *complexity* at which a system is considered.

The concept of complexity as used here does not refer to the degree of randomness that a system exhibits (as in algorithmic complexity). Rather it refers to a number of features, such as nested multi-level relations within a system, (self-referential) mechanisms for sustaining itself, nonlinear interactions among variables, interplay of deterministic and stochastic elements, etc. Measures of complexity in this sense are convex, not monotonic, as a function of randomness: they vanish for purely regular and for purely random behaviour and achieve their maximum somewhere in between [49]. Complexity in this sense can be roughly assumed to increase from simple to less simple inanimate systems over living systems to organisms with the capacity for communication, for understanding meaning and for acting as purposeful agents in their environment.

The basic idea for relevance relations in the context of reproducibility is that each level of complexity is optimally, i.e. most relevantly, related to a particular range of descriptive granularity. This range provides the relevant features for a proper understanding of a system at the level of complexity considered. In this spirit, complexity is not a property of a system as such, but is understood as *relative* to particular selected aspects of the system. Features satisfying the relevance relation are then the objects of choice for empirical investigations designed to further our understanding. And these relevant features need to be focused on for reproducible research.

**Figure 1.**
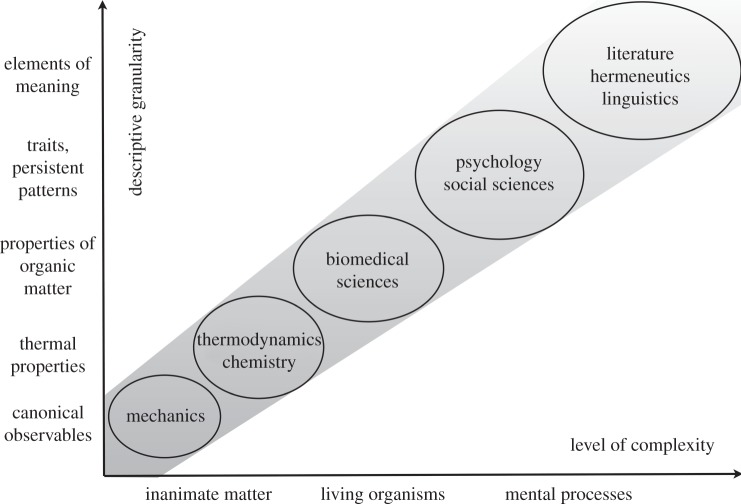
The granularity of a description is illustrated in relation to the level of complexity at which a system is described. Granularity is understood to increase from fine-grained to coarse-grained descriptions, complexity is understood as a convex measure between regularity and randomness (for details see text). The area represented by the blobs characterizes ‘relevance relations’, which indicate the descriptive granularity that is relevant for a particular level of complexity. Note that the same system can be described with different granularity at different levels of complexity. A description of a certain granularity is ‘appropriate’ for a selected level of complexity if it satisfies the relevance relation.

A sketch of how relevance relations might be conceived is illustrated in figure 1. Along the vertical axis, descriptive granularity (increasing from fine-grained to coarse-grained descriptions) is characterized by a selection of properties that figure as candidate examples for relevance. The horizontal axis indicates the level of complexity at which a system is considered from inanimate (physical) matter to living organisms (biomedical science) and further to mental processes for which issues such as meaning and agency become significant.

The series of differently sized blobs gives an idea about which range of granularity might be expected as relevant for which range of complexity. To be sure, the intention of exploiting relevance relations is not to find the ‘correct’ granularity for a ‘given’ system complexity. As mentioned above, a system can be viewed at different levels of complexity and described at different levels of granularity. Empirical or other contexts^16^ decide which levels are to be selected in a given situation. Once a level of complexity is selected, however, relevance relations limit the range of proper descriptive granularity.

The general trend for such relevance relations is an increase in descriptive granularity as complexity increases. For complex systems, the relevant features arise from rather coarse-grained descriptions, whereas for simple systems the relevant features arise from fine-grained descriptions. This implies that irrelevant features of complex systems tend to be too fine-grained, whereas irrelevant features of simple systems tend to be too coarse-grained. In the intermediate range of living organisms, the proper granularity is more difficult to find, because the threat of irrelevance lurks on both sides, too coarse-grained and too fine-grained.

The case of drug development in biomedical sciences is particularly interesting because it includes the problem of how to *translate* results from biochemical laboratory studies to preclinical animal studies and further to clinical studies with humans [51]. The framework of relevance relations as sketched in figure 1 suggests that this translation problem must be addressed (and possibly solved) in (at least) two dimensions. Increasing the level of complexity at which the system is considered from laboratory to preclinical to clinical studies implies that the descriptive granularity required to hit the proper features for reproducibility typically increases as well. In addition, translating biomedical variables such as medication efficacy to higher levels of complexity (from animal to human) has an ‘evolutionary dimension’. Relevant predictions on treatment success for a specific carcinoma require the choice of an animal species that is spontaneously receptive for the same tumour as humans are.

In this regard, *mereology* could provide clues for a proper identification of relevance relations that can be applied to the issue of reproducibility. Mereology is concerned with relationships between parts and wholes (cf. the mereology handbook by Burkhardt *et al*. [52]), which are crucial for assessing the complexity of a system. As wholes typically have (emergent) properties that parts do not have, attempts at reproducing such properties rely on a descriptive granularity properly adjusted to the considered features of a system. Relevance relations provide guidelines for this adjustment. Findlay & Thagard [53] recently proposed a scheme for constitution and emergence with a focus on biological, cognitive and social systems, which may be read accordingly.

### 4.3. Transformations across levels of granularity and complexity

Relations across levels of granularity are a key aspect of sensible interdisciplinary work. Dissent about ‘proper’ levels of granularity appears typically in the process of problem framing during or even at the beginning of interdisciplinary projects. Difficulties arise especially if basic sciences are involved whose firm belief is that subjacent concepts and formalisms govern processes at higher (‘less basic’) levels. This is related to the assumption that finer-grained descriptions contain all information needed for coarser descriptions, which are thus taken as derivable from or reducible to lower level descriptions.

Examples for this overly idealistic view abound, for instance, in personalized medicine. Here, common strategies for drug design require a mechanistic understanding of the human organism at the level of molecular interactions. While single chemical reactions in the cell are quite well understood, their interaction in a chemical network, which eventually leads to cellular responses to a stimulus from outside the cell, is still at the cutting edge of medical research. The ‘personal pill’, a promise of the last decade, is far from breaking through [54].

In addition to reducing human disease to the biochemistry of the cell, the ‘personal pill’ requires an ‘individualization’ of the cellular biochemistry. The dominant view of cellular individualization today is essentially based on bookkeeping of the genes and in part of their products, the proteins. Individual behaviour of cells, tissues and bodies, however, seems to emerge out of a complex interplay of contexts: the tissue context for the cell, the body's context for tissue and organ, the world's context for the body [55]. Carrying this view to extremes, a tumour may be considered as the individualization of a cell whose malign properties result from all kinds of contexts.

In such a contextual picture, questions about ‘the proper’ level of granularity or complexity appear hegemonic rather than constructive. The actual art of interdisciplinary research is not to find ‘one best’ ‘most appropriate’ descriptive granularity of a system, but to combine multi-level approaches in an intelligent way. A pertinent recent example is the novel field of epigenetics. Jablonka & Lamb [56] distinguish genetic, epigenetic, behavioural and symbolic dimensions as four determinants of living systems, each of which can easily be further refined into subdimensions (and so on). We propose that relevance relations are a useful framework for coordinating such different dimensions in an overall coherent picture in which varying levels of granularity and complexity complement rather than exclude one another.

An empirically applicable scheme to relate descriptive levels across disciplines in a formally well-defined fashion was introduced by Bishop & Atmanspacher [57]. The basic idea is that a lower level (fine-grained) description is only necessary, but not sufficient, for higher level (coarser) descriptions. Contingent higher level contexts can be formally implemented to obtain surrogates for the missing lower level sufficient conditions. A review of the conceptual framework of contextual emergence is owing to Atmanspacher & beim Graben [58]. To facilitate readers’ access to the key ideas, appendix A describes how to construct contextually emergent features and outlines examples from physics, chemistry, cognitive neuroscience and the philosophy of mind.

Modern chemotherapy can be regarded as another fitting case in point, waiting to be worked out in detail. Tumour markers have been identified to indicate cancer and to monitor cancer therapy. In this aspect, tumour markers can be viewed as ‘surrogates’ in the sense that a surrogate endpoint of a clinical trial is a laboratory measurement or a physical sign used as a substitute for a clinically meaningful endpoint that measures directly how a patient feels, functions or survives. Changes induced by a therapy on a surrogate endpoint are expected to reflect changes in a clinically meaningful endpoint.

As can be seen from these examples, and from many others in modern biomedicine, well-designed interdisciplinary projects are characterized by carefully designed transformation strategies and procedures across levels of granularity and complexity. It is absolutely necessary to permanently reflect and translate the meaning of a ‘surrogate’ for the object (or subject) under inspection. A proper concept for interlevel relations is the touchstone of successful interdisciplinary work, distinguishing it from merely additive multi-disciplinary collaborations.

## 5. Ontological relativity: beyond fundamentalism and relativism

A network of descriptive levels of varying degrees of granularity raises the question of whether descriptions with finer grains are more ‘fundamental’ than those with coarser grains. The majority of scientists and philosophers until today have tended to answer this question affirmatively. As a consequence, there would be one fundamental ontology, preferentially that of elementary particle physics, to which the terms at all descriptive levels can be reduced.

But this reductive credo has also received critical assessments and alternative proposals.^17^ A philosophical precursor of trends against a fundamental ontology is Quine's [60] ‘ontological relativity’ (carrying Garfinkel's ‘explanatory relativity’ into ontology). Quine argued that if there is one ontology that fulfils a given descriptive theory, then there is more than one. In other words, it makes no sense to say what the objects of a theory are, beyond saying how to interpret or reinterpret that theory in another theory.

For Quine, any question as to the ‘quiddity’ (the ‘whatness’) of a thing is meaningless unless a conceptual scheme is specified relative to which that thing is discussed. For Quine, the inscrutability of reference (in combination with his semantic holism) is the issue which necessitates ontological relativity. The key motif behind it is to allow ontological significance for any descriptive level, from elementary particles to ice cubes, bricks and tables, and further to thoughts, intentions, volitions and actions.

Quine proposed that the ‘most appropriate’ ontology should be preferred for the interpretation of a theory, thus demanding ‘ontological commitment’. This leaves us with the challenge of how the ‘most appropriate’ should be defined, and how corresponding descriptive frameworks are to be identified. Here is where the idea of relevance relations becomes significant. For a particular level of complexity in a given context, the ‘most appropriate’ framework is the one that provides the granularity given by the relevance relation. And the referents of this descriptive framework are those which Quine wants us to be ontologically committed to.

On the basis of these philosophical approaches, Atmanspacher & Kronz [61] suggested the concept of ‘relative onticity’ as a way of applying Quine's ideas to concrete scientific descriptions, their relationships with one another and with their referents. One and the same descriptive framework can be construed as either ontic or epistemic, depending on which other framework it is related to: bricks and tables will be regarded as ontic by an architect, but they will be considered highly epistemic from the perspective of a solid-state physicist.

This farewell to the centuries-old conviction of an absolute fundamental ontology (usually that of basic physics) is still opposed to modern mainstream thinking today. But in times in which fundamentalism—in science and elsewhere—appears increasingly tenuous, ontological relativity offers itself as a viable alternative for more adequate and more balanced frameworks of thinking. And, using the scientifically tailored concepts of relative onticity outlined above, it is not merely a conceptual idea but can be applied for an informed discussion of concrete scenarios in the sciences.

Coupled with an ontological commitment that becomes explicit in relevance relations, the relativity of ontology must not be confused with dropping ontology altogether. The ‘tyranny of relativism’ (as some have called it) can be avoided by identifying relevance relations to select proper context-specific descriptions from less proper ones. The resulting picture is more subtle and more flexible than an overly bold reductive fundamentalism, and yet it is more restrictive and specific than a patchwork of arbitrarily connected opinions.

## Endnotes

1 These references are a tiny subset of the existing literature on problems with reproducibility. Many more examples will be discussed in the main body of this article.

2 This tenet was challenged by Popper [6], whose main point of criticism was due to a switch of perspective from ahistoric, time-independent knowledge to historically evolving knowledge. While it most often turns out inconsequential to disregard historicity in basic science, its consideration is crucial in behavioural, social and cultural science. Therefore, it is not astonishing that most early literature on reproducibility is found in these areas. Classic monographs are Sidman [7], Krathwohl [8] or Collins [9].

3 See Peres [10, ch. 12] for an informed (yet somewhat technical) discussion of measurement in quantum physics and its peculiarities, which gave rise to a number of stronger or weaker versions of scientific anti-realism. Of particular interest (see §4) is the variant proposed by van Fraassen [11], reviewed by Monton & Mohler [12].

4 Procrustes is a bandit in Greek mythology who preyed on travellers by inviting them for dinner and then stretching or chopping them until they exactly fit his bed. Eddington [13] used the figure of Procrustes to illustrate his concept of ‘selective subjectivism’: he invented an apocryphal paper by Procrustes ‘On the uniform length of travellers’, submitted to the Anthropological Society of Attica. See also Oldroyd [14, pp. 281–284].

5 This does not cover the case of statistical laws in quantum physics, which needs separate consideration. But this would exceed the scope of this cursory sketch.

6 blogs.plos.org/everyone/2012/08/14/plos-one-launches-reproducibility-initiative/

7 openscienceframework.org/project/EZcUj/wiki/home

8 In his *Philosophical Investigations*, par. 109, Wittgenstein states: ‘Philosophy is a battle against the bewitchment of our intelligence by language’.

9 By the way, a particular technique of multivariate statistics is ‘Procrustes analysis’, a least-squares method to perform simultaneous affine transformations (translation, rotation, scaling) among matrices up to their maximal agreement. Originally introduced by Schönemann [32] in psychology, the method ‘massages’ data such that subsets of maximal similarity in shape and size can be distinguished. For an overview see Gower & Dijksterhuis [33].

10 Kahneman wrote (see [36] for the full letter): ‘The storm of doubts is fed by several sources, including the recent exposure of fraudulent researchers, general concerns with replicability that affect many disciplines, multiple reported failures to replicate salient results in the priming literature, and the growing belief in the existence of a pervasive file drawer problem that undermines two methodological pillars of your field: the preference for conceptual over literal replication and the use of meta-analysis. Objective observers will point out that the problem could well be more severe in your field than in other branches of experimental psychology, because every priming study involves the invention of a new experimental situation. For all these reasons, right or wrong, your field is now the poster child for doubts about the integrity of psychological research’.

11 In those years, the sheer presumptions of the neurosciences reached an unprecedented climax with claims such as turning theology into neuro-theology or economics into neuro-economics.

12 The notion of *mimesis* has also acquired significance in psychoanalysis [40] and in recent approaches covered by the notion of ‘theory of mind’ [41]. However, this discussion is beyond the scope of the present account.

13 Today it is also questionable that, as proposed by the ‘generative grammar’ of Chomsky, meaning derives from a purely syntactic deep structure of language.

14 On p. 127, footnote 34, van Fraassen [11] acknowledges that this ‘idea was independently developed by … Alan Garfinkel in *Explanation and Individuals* (Yale University Press, forthcoming)’. Obviously, he is referring to a preliminary version of Garfinkel [44].

15 The significance of context is already indicated in Weaver's commentary to Shannon's purely syntactic theory of communication [46]. It is an essential part of the concept of pragmatic information proposed by von Weizsäcker [47].

16 Recent work by Peschard & van Fraassen [50], in which they address the issue of relevance judgments in experimental modelling (‘data-generating procedures’), discusses the extension of such contexts to norms and values.

17 An informative critique of fundamental particle ontologies is due to Bitbol [59].

## Appendix A. Contextual emergence

The basic goal of contextual emergence is to establish a well-defined interlevel relation between a ‘lower’ level *L* and a ‘higher’ level *H* of a system. This is done by a two-step procedure that leads in a systematic and formal way (i) from an *individual* description *L_i_* to a *statistical* description *L_s_* and (ii) from *L_s_* to an individual description *H_i_*. This scheme can in principle be iterated across any connected set of descriptions, so that it is applicable to any situation that can be formulated precisely enough to be a sensible subject of scientific investigation.^18^

The essential goal of step (i) is the identification of equivalence classes of individual states that are indistinguishable with respect to a particular ensemble property. This step implements the multiple realizability of statistical states in *L_s_* by individual states in *L_i_*. The equivalence classes at *L* can be regarded as cells of a partition. Each cell is the support of a (probability) distribution representing a statistical state, encoding limited knowledge about individual states.

The essential goal of step (ii) is the assignment of *individual* states at level *H* to *statistical* states at level *L*. This cannot be done without additional information about the desired level-*H* description. In other words, it requires the choice of a *context* setting the framework for the set of *observables* (properties) at level *H* that is to be constructed from level *L*. The chosen context provides constraints that can be implemented as *stability* criteria at level *L*. It is crucial that such stability conditions cannot be specified without knowledge about the context at level *H*. In this sense, the context yields a top-down constraint or downward confinement (sometimes misleadingly called downward causation).

The notion of stability induced by context is of paramount significance for contextual emergence. Roughly speaking, stability refers to the fact that some system is robust under (small) perturbations. For example, (small) perturbations of a homoeostatic or equilibrium state are damped out by the dynamics, and the initial state will be asymptotically retained. The more complicated notion of a stable partition of a state space is based on the idea of coarse-grained states, i.e. cells of a partition whose boundaries are (approximately) maintained under the dynamics.

Such stability criteria guarantee that the statistical states of *L_s_* are based on a robust partition so that the *emergent* observables in *H_i_* are well defined. Implementing a contingent context at level *H* as a stability criterion in *L_i_* yields a proper partitioning for *L_s_*. In this way, the lower level state space is endowed with a new, *contextual topology*. From a slightly different perspective, the context selected at level *H* decides which details in *L_i_* are relevant and which are irrelevant for individual states in *H_i_*. Differences among all those individual states at *L_i_* that fall into the same equivalence class at *L_s_* are irrelevant for the chosen context. In this sense, the stability condition determining the contextual partition at *L_s_* is also a *relevance condition*.

This interplay of context and stability across levels of description is the core of contextual emergence. Its proper implementation requires an appropriate definition of individual and statistical states at these levels. This means in particular that it would not be possible to construct emergent observables in *H_i_* from *L_i_* directly, without the intermediate step to *L_s_*. It would be equally impossible to construct these emergent observables without the downward confinement arising from higher level contextual constraints.

In this spirit, bottom-up and top-down strategies are interlocked with one another in such a way that the construction of contextually emergent observables is *self-consistent*. Higher level contexts are required to implement lower level stability conditions leading to proper lower level partitions, which in turn are needed to define those lower level statistical states that are co-extensional (not necessarily identical!) with higher level individual states and associated observables.

The procedure of contextual emergence has been shown to be applicable to a number of examples from the sciences. Paradigmatic case studies are: (i) the emergence of thermodynamic observables, for example temperature, from a mechanical description [57], (ii) the emergence of hydrodynamic features, for example Rayleigh–Bénard convection, from many-particle theory [62] and (iii) the emergence of molecular observables, for example chirality, from a quantum mechanical description [57,63].

If descriptions at *L* and *H* are well established, as is the case in these examples, formally precise interlevel relations can be straightforwardly set up. The situation becomes more challenging, though, when no such established descriptions are available, e.g. in cognitive neuroscience or consciousness studies, where relations between neural and mental descriptions are considered. Even there, contextual emergence has been proved viable for the construction of emergent mental states [64,65] and an informed discussion of the problem of mental causation [66].
